# Medicine Prices, Availability, and Affordability in Private Health Facilities in Low-Income Settlements in Nairobi County, Kenya

**DOI:** 10.3390/pharmacy7020040

**Published:** 2019-04-24

**Authors:** Dennis Ongarora, Jamlick Karumbi, Warnyta Minnaard, Kennedy Abuga, Vincent Okungu, Isaac Kibwage

**Affiliations:** 1 Department of Pharmaceutical Chemistry, University of Nairobi, Nairobi 19676-00202, Kenya; koabuga@gmail.com (K.A.); ikibwage@gmail.com (I.K.); 2 Ministry of Health, Nairobi 30016-00100, Kenya; karumbij@gmail.com; 3 Stichting PharmAccess International, 22700 1100 DE Amsterdam, the Netherlands; w.minnaard@pharmaccess.org; 4 PharmAccess Foundation, Nairobi 6711-00100, Kenya; okungu008@gmail.com; 5 Administration, Planning and Development, Egerton University, Njoro 20115, Kenya; ikibwage@gmail.com

**Keywords:** retail medicine prices, availability, affordability, low-income settlements, international reference price

## Abstract

Medicine prices are a major determinant of access to healthcare. Owing to low availability of medicines in the public health facilities and poor accessibility to these facilities, most low-income residents pay out-of-pocket for health services and transport to the private health facilities. In low-income settlements, high retail prices are likely to push the population further into poverty and ill health. This study assessed the retail pricing, availability, and affordability of medicines in private health facilities in low-income settlements within Nairobi County. Medicine prices and availability data were collected between September and December 2016 at 45 private healthcare facilities in 14 of Nairobi’s low-income settlements using electronic questionnaires. The International Medical Products Price Guide provided international medicine reference prices for comparison. Affordability and availability proxies were calculated according to existing methods. Innovator brands were 13.8 times more expensive than generic brands. The lowest priced generics and innovator brands were, on average, sold at 2.9 and 32.6 times the median international reference prices of corresponding medicines. Assuming a 100% disposable income, it would take 0.03 to 1.33 days’ wages for the lowest paid government employee to pay for treatment courses of selected single generic medicines. Medicine availability in the facilities ranged between 2% and 76% (mean 43%) for indicator medicines. Prices of selected medicines varied within the 14 study regions. Retail medicine prices in the low-income settlements studied were generally higher than corresponding international reference prices. Price variations were observed across different regions although the regions comprise similar socioeconomic populations. These factors are likely to impact negatively on healthcare access.

## 1. Introduction

Governments in low- and middle-income countries (LMICs) are increasingly advocating for universal health coverage [1]. One of the key components of any functional health system is the availability of medicines [2]. However, a majority of the population in LMICs have limited access to medicines [3]. Even when medicines are available, patients have to pay for them out-of-pocket, the cost contributing up to 60% of health care expenditure [4]. Access to medicine is broadly defined by proximity to health facilities, affordability, availability, and acceptability. Affordability is heavily dependent on the price of medicines, while availability is usually linked to the demand for the medicines [5]. In many LMICs, the majority of the urban population resides in informal low-income settlements or slums [6] and their disposable income is often meagre thus affecting affordability of medicines [7].

The capital of Kenya, Nairobi, is home to a number of low-income settlements including Mukuru, Kawangware, Kibera, and Mathare [8]. The physical environment in slums, including the lack of access to safe drinking water, poor sewerage drainage, and haphazard garbage disposal, are major contributors to the observed poor health status in slums. Other factors such as poor socio-economic conditions, overcrowding, and inadequate health and social services also play a role [9,10].

Slum residents earn their living by working as casual laborers, street vendors, or petty shop operators and barely make enough to meet their basic needs, including healthcare [11,12,13]. A 2005 report constructed from national health accounts observed that more than a third of poor patients in Kenya did not seek medical attention [14]. Studies have indicated that patients in low-income settlements categorize diseases into those that should be attended to urgently and those that can wait because of monetary constraints [15].

Amuyunzu-Nyamongo and Nyamongo reported that mothers of ill children under-five-years old in Nairobi slums visited retail shops as their first stop and only visited health facilities when the illness persisted. The respondents in the study preferred private facilities to public ones citing the proximity, flexible payment options, and perceived better quality of medicines and services offered [15]. Public health facilities, the respondents felt, were characterized by shortages of medicines and other supplies as well as discourteous workers [15]. Retail pharmacies in the slums are increasingly recognized as the first and sometimes the only contact for the urban poor with conventional medicine [16]. This observation holds true for other low-income countries [17,18].

In Kenya, the government partially subsidizes the cost of healthcare for all its citizens mainly in public facilities. With donor support, programs for the management of HIV/AIDS, TB, STIs, and maternal and child health including childhood vaccination in the public sector can be said to be robust. However, government funding for the management of other acute and chronic conditions is inadequate. Public facilities are plagued with low availability of medicines and laboratory supplies [19]. Low morale among public healthcare staff as evidenced by frequent strikes against poor remuneration and poor working conditions also hinders the optimal delivery of healthcare in public health facilities [20]. Furthermore, low-income settlements are prone to diseases associated with poor sanitation and crowding [21]. The situation in these settlements is exacerbated by the availability of few public health facilities to serve the large population. There is no agreed operating framework of operation between private and public health facilities. Patients are free to choose which facility to visit. The government regulates the registration of medicines, health facilities, and health personnel, but not medicine prices. Health facilities (both public and private) in low-income settlements are generally manned by nursing and paramedical staff and tend to be cheaper than similar facilities in more affluent settlements.

Private and public insurance schemes complement government healthcare funding for about 25% of the population [22]. In 2014, only 11% of the Kenyan population was covered by the affordable government-controlled National Hospital Insurance Fund (NHIF). NHIF coverage within the informal workforce was low, at only 16% [23]. Thus, most Kenyans incur their healthcare costs privately and mainly through out-of-pocket (OOP) payments. For example, in the financial year 2014/15, a national health account report indicated that about 40% of total health expenditure, the largest single source, came from private sources. Of these sources, OOP payments contributed 65% of the total for low-income populations such as the ones studied here [24]. This observation can be attributed to limited and irregular earnings in this population segment. On the other hand, in 2016, OOP payments constituted the single largest component of the total health expenditure in Kenya at about 28% [24].

The cost of medicines accounts for 20–60% of healthcare costs in developing countries [4]. Thus, prices of essential medicines that are disproportionately higher than available income contribute significantly to morbidity and mortality, particularly in low-income settlements. Few comprehensive studies on medicine prices have been carried out in Kenya with the 2006 medicine price survey by the WHO standing out [25]. Previous studies on medicine prices report national data. Such studies do not take into account the large divide between the poor and the affluent in developing countries. This study focused on a low-income population.

The purpose of this study was to assess medicine pricing, affordability, and availability in low-income settings. A better understanding of medicine pricing challenges will feed the debate on possible remedies.

## 2. Materials and Methods

### 2.1. Study Design

The medicine prices, affordability, and availability study was nested in a larger cross-sectional descriptive study conducted between September and December 2016 in Nairobi County, Kenya. The study targeted private healthcare providers in low-income settlements. A standardized electronic questionnaire was used for data collection to gather information from the healthcare providers on inventory management practices.

### 2.2. Study Area Description

**Figure 1:**
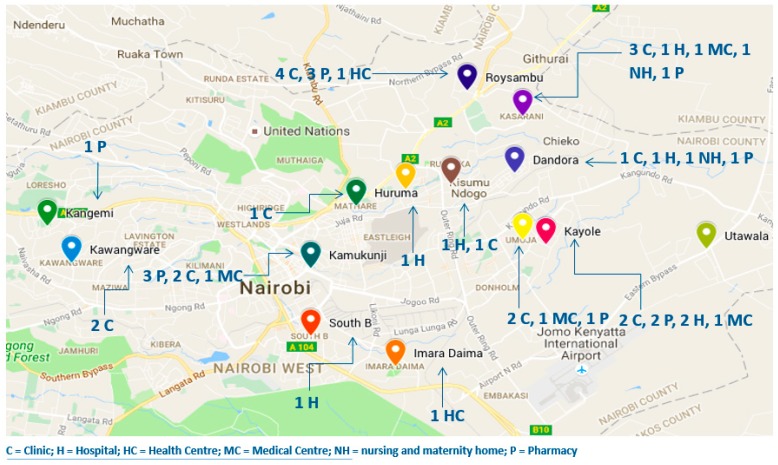
Map of Nairobi County showing study sites.

Nairobi County is divided into nine sub counties, of which five (Dagoretti, Starehe, Embakasi, Kamukunji, and Kasarani) were targeted for this study as these sub counties are home to some of the biggest slum dwellings in the country and inhabited by many people earning less than $1.25 per day [26]. The approximate geographical locations of the participating facilities are illustrated in Figure 1.

### 2.3. Study Sites and Sampling

The study population consisted of a total of 232 healthcare providers (health facilities and standalone pharmacies) in Nairobi County enrolled with various m-Health interventions and that form a part of the PharmAccess Foundation-Kenya healthcare provider database that serves low-income patients. From the pool of 232 facilities, 91 conformed to the inclusion/exclusion criteria. To be included in the study, healthcare facilities had to be located in a low-income urban settlement, licensed, and involved in the purchase and sale of medicines to low-income patients. Health facilities within Kibera slums were not included because of the evidence that several NGOs and programs providing free healthcare services operate in this settlement [Population Council, 2009]. Fifty facilities were recruited through purposive sampling with 45 facilities (18 clinics, 7 hospitals, 2 health centres, 4 medical centres, 2 nursing and maternity homes, 12 pharmacies) spread in five sub counties consenting to participate in the study.

### 2.4. Data Collection and Data Management

Trained field agents used a structured, standardized electronic questionnaire to capture data from the 45 healthcare providers on the medicines they had in stock. Data collected included healthcare provider type; formulation type, dose, strength, pack size, and retail price of drug molecule; manufacturer; therapeutic class; number of products in stock, Kenya Essential Medicine List Category Number; whether the medicine was an innovator brand or generic; and the product name. The project pharmacist and project manager validated and anonymized the data by assigning unique identifiers in the database and reports to maintain confidentiality. Data was entered and cleaned using Microsoft Excel 2010 (Microsoft, Seattle, WA, USA).

### 2.5. Data Analysis

Thirty-five drug molecules captured during the survey were analyzed. The selection of the molecules was based on inclusion in previous WHO price comparison surveys [19] and significance based on the local disease burden in the area under study. Further data cleaning and analysis was conducted using STATA version 13 [27]. The unit price was obtained by dividing the retail price per pack by the pack size.

Analysis of price, availability, and affordability was carried out. Univariate analysis was performed to determine the price variation (median, interquartile range, and range) for both innovator and generic brands. For the generic brands, the data was summarized for the lowest priced generic (LPG) and for the most sold generic (MSG). International reference prices (IRPs) for 2015 were obtained from The International Medical Products Price Guide, published by Management Sciences for Health. The International Medical Products Price Guide details prices for generic medicines obtained from pharmaceutical suppliers, international development organizations, and government agencies in different countries and gives a median price for each product listed [28]. The IRPs were used in the calculation of the median price ratios (MPR), the ratio of the median price of a given medicine to the corresponding IRP. An MPR of 1.0 indicates that the price of the medicine in the study area is the same as the international reference price. Values greater than 1.0 indicate that the medicine price is higher in the study area as compared to the international reference price.

Affordability was assessed by calculating the number of days it would take the lowest paid government worker (LPGW) to buy a course of treatment for selected medicines [4]. Medicines used to manage three chronic and two acute conditions that are adjudged highly prevalent in the low-income settlements were included. For each condition, lowest priced generic costs of selected alternative drug treatment regimens were computed and compared. Affordability was calculated by dividing the total cost of a treatment regimen by USD 5.27 (527.10 Kenyan shillings), the minimum daily wage for cleaners, gardeners, general workers, house servants, children’s nannies, sweepers, day watchmen, and messengers in the year 2015/2016 for Nairobi [29]. These are unskilled workers who are likely to be residing in low-income settlements.

Availability was calculated as “the percentage of facilities in which the medicine was found on the day of data collection” [30]. A basket of 15 medicines included in more than 80% of WHO surveys was adopted for this purpose [4].

## 3. Results

### 3.1. Comparison of Innovator Brand, Lowest Price Generic Medicines, and International Reference Prices

The median prices of the innovator brand (IB) and lowest priced generic (LPG) are tabulated in Table 1. Included in the same table are the international reference prices (IRP), the ratio of the IB price to the price of the LPG as well as the median price ratios (MPR) for the IB and LPG.

Innovator products were available for 28 products selected for inclusion in the study. Of the 28, only salbutamol inhaler had a median lowest generic price higher than the price of the innovator brand. The other 27 innovator products were 1.5 to 76.3 times more expensive than the corresponding LPGs. Seven innovator products, namely levonorgestrel (1.5), amoxicillin/clavulanic acid 625 mg (2.5), paracetamol 500 mg (3.2), albendazole suspension (4), ranitidine 5 mg (5), cetirizine 10 mg (5), and glibenclamide 5 mg (5) were sold at 1–5 times the price of the LPG equivalents. Eight innovator products were sold at between 5–10 times the price of the corresponding LPGs. Six innovator products were 10 to 20 times more expensive than the LPGs, while six products, namely, omeprazole 20 mg (20), amoxicillin 500 mg (25), chlorpheniramine 4 mg (30), metronidazole suspension (30), ciprofloxacin 500 mg (35.0), and ceftriaxone injection 1g (76.3) were ≥20 times more expensive than the corresponding LPGs. On average, IBs were 13.8 times more expensive than the LPGs.

Of the 35 products for which IRPs were available, only eight products (22.9%) (artemether/lumefantrine 20 mg/120 mg, amoxicillin/clavulanic acid 250/62.5 mg/5 mL suspension, amoxicillin 500 mg, metronidazole 200 mg/5 mL suspension, metformin 500 mg, ibuprofen 400 mg, metronidazole 400 mg, and metronidazole 400 mg) had an LPG MPR <1 implying that they were cheaper in the study area compared to the IRPs. The LPG MPR ranged between 1.0 (paracetamol 500 mg) and 11.6 (hydrochlorthiazide 25 mg) for the remaining 24 products. The population in the study area was thus paying higher generic prices than the median IRPs for 68.6% of the generic medicines selected. The six products whose LPGs were >5 times the corresponding median IRPs included glibenclamide 5 mg, diclofenac 50 mg, omeprazole 20 mg, cetirizine 10 mg, atenolol 50 mg, and hydrochlorothiazide 25 mg. The average LPG MPR was 2.9 for all the 35 products.

The IB MPR was calculated for 28 innovator products and ranged from 1.0 (artemether/lumefantrine) to 150.0 (chlorpheniramine). Fourteen products (50%) had MPRs of >10, six of them with MPRs of >50. Diclofenac, omeprazole, and chlorpheniramine had IB MPRs of >100. On average, the IB MPR for the 35 products was 32.6.

### 3.2. Price Variation across the Regions during the Study Period

**Figure 2:**
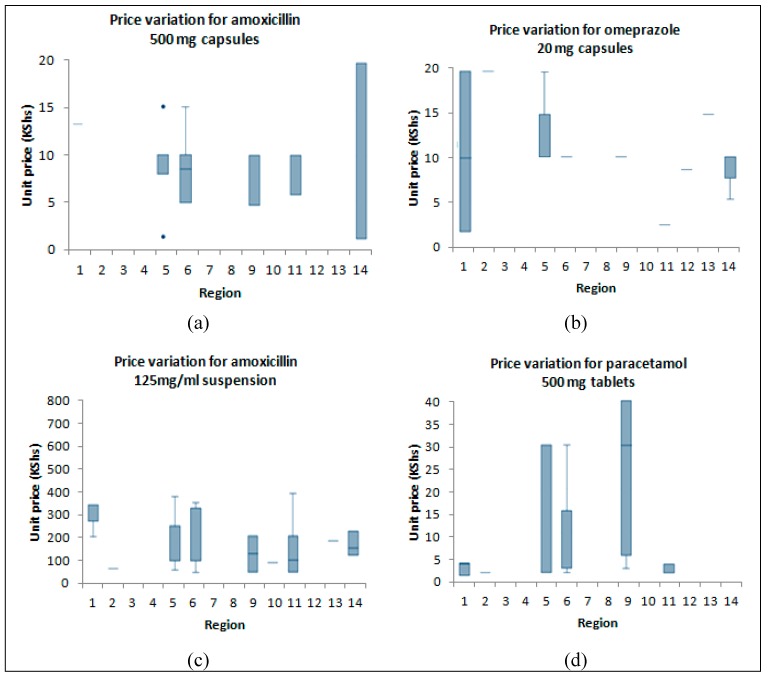
Price variations for selected medicines across regions. **Key**: Prices are in KShs (100 KShs ≈ 1 USD). The edges of each box represent the lower and upper quartiles while the median prices are represented by the horizontal lines crossing the bars. The outer lines (whiskers outside the box) represent the highest and lowest observation. The isolated points are outlier prices. The regions in the study were 1 Dandora, 2 Eastleigh, 3 Huruma, 4 Imara Daima, 5 Jogoo Road, 6 Kahawa West, 7 Kangemi, 8 Kariobangi, 9 Kasarani/Mwiki, 10 Kawangware/Dagoretti, 11 Kayole, 12 Mathare, 13 South B, and 14 Umoja.

The prices of four of the most widely available products (amoxicillin 500 mg capsules, amoxicillin 125 mg/mL suspension, paracetamol tablets, and Omeprazole 20 mg capsules) were compared across the various regions included in the study. The data is graphically displayed in Figure 2. Inter-regional variations in median prices were observed for all products.

### 3.3. Comparison of Prices within Therapeutic Classes

The prices for alternative treatments (LPGs) within selected therapeutic classes are shown in Table 2. Existing standard treatment regimens were used [31]. Among the anti-ulcer medicines, 30-day courses of omeprazole 20 mg and ranitidine 300 mg were virtually equally priced. Among the anti-bacterial medicines, a 7-day course of amoxicillin was the cheapest (seven times cheaper than the next cheapest antibacterial, ciprofloxacin). A 7-day amoxicillin/clavulanate 625 mg course was the highest priced antibacterial, A 30-day furosemide course was the cheapest of the five anti-hypertensives selected, while atenolol and enalapril courses were the most expensive in this class. Within the anti-diabetic class, the 30-day metformin 500 mg course was one third as costly as a 30-day course of glibenclamide.

### 3.4. Affordability

The retail prices for the different treatment courses in Table 2 were applied in the standard method of estimating affordability based on how many days it would take the lowest paid government worker (LPGW) to purchase a given treatment course.

Assuming all wages are used for buying drugs, it would take less than a day to purchase all the selected drugs except amoxicillin/clavulanic acid 625 mg, which would require 1.33 days’ wages to pay for a 7-day course. It would take 0.76 days to buy a salbutamol inhaler used in the treatment of asthma. To buy a basket consisting of a course of amoxicillin/clavulanic acid for an infection, a one-month course of nifedipine for hypertension and a salbutamol inhaler, a worker would spend 2.37 days’ wages. A basket comprising a salbutamol inhaler and a course of cotrimoxazole suspension for a child with a respiratory tract infection would consume 1.52 days’ wages.

For comparison purposes, other affordability methods assume disposable incomes of 7% and 40% for healthcare expenditure [32,33]. Using these methods, the number of days’ wages required to pay for a given medicine would go up by 14.29 and 2.50 times, respectively. Assuming 7% disposable income, we note that the affordability of common medicines drops drastically as evidenced by the number of days the LPGW would have to work to purchase typical treatment courses. For example, to purchase a course of amoxicillin/clavulanic acid 625 mg, it would require 19 days’ wages.

### 3.5. Availability of Medicines in the 45 Facilities

The availability of the basket of 15 medicines found in at least 80% of previous WHO surveys is presented in Table 3.

The basket of 15 medicines adopted from previously conducted availability studies includes captopril. However in this survey, none of the 45 facilities stocked captopril [4]. Enalapril was the more frequently encountered of the only two angiotensin converting enzyme inhibitors (ACEI) in stock, and was found in 44% of all facilities. The other ACEI was lisinopril. Six medicines (ceftriaxone, hydrochlorothiazide, omeprazole, sulfamethoxazole/trimethoprim, amoxicillin, and ciprofloxacin) were available in more than 60% of facilities. Availability was less than 50% for the remaining nine medicines. The average availability for the basket of medicines was 43%. Beclomethasone inhaler was only available in one facility (2% availability). The retail price for the generic inhaler was KShs 855 (US$ 8.55), making it quite costly. Fluoxetine tablets were available in only two facilities (4% availability). The only other medicine with less than 20% availability was amitriptyline which was found in eight facilities (18% availability).

## 4. Discussion

The results in this study show that, overall, generic medicines are sold at lower retail prices than innovator brands. The large price difference between the generic and innovator brands observed is similar to the findings of a 2006 WHO study of medicine prices in Kenya [34], which reported IB:LPG ratios in the private sector of 7.5 to 50 for the ten medicines with the greatest price differences. However, the average IB:LPG ratio in the WHO study was 5.09 [34], while that obtained in this study was 13.8. Although this data confirms that generic products were generally much cheaper than innovator products, the large price differentials observed appear grossly exaggerated for the target poor population. In cases where some patients may prefer to purchase the innovator product, affordability would be greatly affected by the high prices. It has been reported that, compared to better educated and more prosperous patients, low-income patients are more likely to associate expensive medicines with increased efficacy and quality [35]. The large price difference between generic and innovator products in this study is in contrast to the findings by Shafie and Hassali in Malaysia, who reported that innovator products were 50% to 90% more expensive than generic medicines [36]. Similarly, the study of medicine prices in 36 countries by Cameron reported that in India the innovator products are only 6% more expensive than generics but this figure is as high as 1465% in LMICs [4].

This study found that LPGs and IBs were 2.9 and 32.6 times, respectively, more expensive than the international reference price for the products analyzed. The 2006 WHO medicine price survey of private facility medicine prices in Kenya reported figures of 3.33 and 17.75 times for LPGs and IBs, respectively, for 45 largely similar products surveyed [34]. Gelders in the 2006 report suggested, as a discussion guideline, that for private retail pharmacies, an MPR <2.5 would represent excessively high local retail prices [37]. Based on this guideline, 12 out of 35 generic medicines (34.3%) selected in the study area may be considered to be excessively priced. A study by Rockers on affordability of non-communicable disease medicines in Kenya concluded that the poorest pay higher prices for medicines than those who are less poor [38].

Artemether/lumefantrine had the lowest MPR (0.2). This may be due to the fact that the Kenyan government has subsidized artemether/lumefantrine in a bid to reduce malaria associated mortality. This is a good example of how government policy can improve the affordability of medicines. The use of median IRPs is, however, associated with a number of limitations, chief among them being the fact that for a number of medicines very few international prices are available. In such cases, the IRP may be skewed to a high or low IRP [4].

Inter-regional price variations were observed among the four selected commonly sold products. This may be attributed to the differential stocking of medicine brands in different localities. Secondly, it may be due to the unregulated pricing adopted by the health outlets. Mark-ups for medicines are not regulated in Kenya and it is possible to obtain the same product from one outlet at several times the price in the next-door outlet. Indeed, Cameron et al. [4] found a wide variation in wholesale and retail mark-ups in their study, especially in the private sector, and recommended that a range of policy options including regulating mark-ups could address price variations. Other problems facing pricing are the lack of a widely accessible database of prices and the procurement of only a few products to avoid dead stock. The latter problem minimizes the discounts that the suppliers are willing to give. From a patient’s perspective a common procurement system as well as standardized and transparent pricing (probably involving the use of digital and mobile platforms) would be highly desirable. The creation of a large cooperative network of facilities is the best way to leverage on economies of scale, access discounts, and minimize price variations. The adoption of centralized procurement among private facilities will require a model that clearly demonstrates advantages to patients as well as health facilities.

Affordability results calculated using the commonly used LPGW method [30,34,39,40] indicated that single LPG medicines were generally affordable in the low-income settlements. Among the medicines considered, a 7-day amoxicillin/clavulanic acid course was the only medicine that the LPGW would have to purchase with more than a day’s wage. Our findings show improved affordability when compared to studies conducted much earlier [34,39,40], but tend to agree with recent findings in Swaziland [30]. Although easy to use, the LPGW method is not an accurate reflection of reality. It is well known that most people in the areas studied earn much less than the lowest paid government employee. For example, a 2016 study reported that 62.9% of the respondents in Mathare, one of the low-income settlements in Nairobi, earned salaries far below the government gazetted minimum wage [41]. It is estimated that 40% of Kenyans live below the poverty line (US$ 1.90 per day) [42]. The percentage of the population living below the poverty line is higher in the slums, estimated at 73% back in 2006 [43]. Furthermore, low-income earners usually have to first pay for housing, food, transport, and other utilities leaving only a small amount available for healthcare. Mokaya et al. have calculated affordability using a method that postulates, based on statistical models, that low-income earners are likely to spend 93% of their income on food, housing, transport, utilities, and sport or leisure activities [32]. Thus, only about 7% of income is available for healthcare costs including the purchase of medicines. Elsewhere, Xu et al. base their affordability calculation on “40% of income remaining after subsistence needs have been met” [33]. Either approach demonstrates that it would take a patient many more days’ wages than estimated by the LPGW method to purchase a course of treatment. Affordability is further compromised by the fact that a patient may need more than one medicine for a particular condition, or someone may be suffering from more than one illness or more than one family member may be unwell. The review by Niëns and Brouwer clearly captures the challenges of operationalizing the concept of affordability and notes that the methods commonly used, including the LPGW, are fraught with limitations [44]. Low affordability would drive patients to forgo treatment thus increasing morbidity and mortality. Patients may also be forced to incur debt or do away with some of their basic needs thus affecting their quality of life.

Availability of medicines in facilities is an important contributor to access. Essential medicines should be readily available. Stock-outs are likely to result in incomplete treatment, treatment disruption, or the use of more expensive alternatives. In this study, we observed that captopril, an antihypertensive medicine, is no longer stocked in private facilities in the study area, seemingly having been replaced by enalapril. Enalapril has been shown to be more potent, longer acting, and possibly safer than captopril [45]. Antibacterial medicines (ceftriaxone, sulfamethoxazole/trimethoprim, amoxicillin, and ciprofloxacin) were available in more than 60% of the facilities. This may be attributed to the fact that infectious diseases are highly prevalent in slums. A survey by Ndugwa and Zulu found that respiratory and gastrointestinal ailments accounted for 46% and 35%, respectively, of the reported symptoms of ill health among respondents in a low-income setting [46]. Fluoxetine and amitriptyline (antidepressants) had very low availability. This may result from the strict control imposed on psychotropic and narcotic substances by law. Furthermore, self-medication for mental disorders is not anticipated. Beclomethasone inhaler was available in only one facility, probably due to the high unit price compared to salbutamol inhaler, which could be obtained at less than half the price.

The WHO medicine price surveys carried out in the early 2000s across Africa (see for example [34,39,40]) and in other parts of the world were meant to identify the reasons for high and variable medicine prices and suggest strategies to improve medicines affordability. Some of the recommendations from these studies included the development and implementation of a “medicines pricing policy to achieve a greater level of transparency, uniformity, and predictability in the pricing of medicines including the consideration of reference pricing for medicines in the private sector”, empowering consumers through dissemination of price information, and adopting pro-poor interventions in essential medicines pricing [34]. Clearly, those recommendations have not been implemented. There is hardly any experience with medicine price controls in developing countries. However, studies have shown that measures such as enhancing generic competition, encouraging local production of generic drugs, and strengthening pharmaceutical regulation may be useful [47,48].

Information and communication technology is widely employed for services ranging from mobile money payments to accessing government services and can be employed to disseminate health information including medicine prices. One approach is the use of mobile apps that link to regularly updated online databases (for example www.pharmafinder.co.ke which lists recommended retail and wholesale prices for several medicines). Targeted education, tailored and simplified for patients with limited knowledge about fair pricing of quality medicines is essential. This can, for example, be achieved through mass text messaging alerting patients of existing price resources.

This study had a number of limitations: medicine prices from facilities in affluent areas in Nairobi were not available for comparison; the LPGW method, although widely used in affordability studies, has inherent weaknesses; there are few recent regional studies to enable reasonable comparisons; and the sample size and study area were limited by the funding available.

## 5. Conclusions

Medicines in the low-income settlements studied were highly priced compared to international reference prices. There were large price differences between generic medicines and innovator products. Although the socioeconomic conditions of the area under study are similar, there were obvious price variations from one location to another. These findings call for the implementation of policies such as the ones recommended by the WHO to address the challenges associated with access to and affordability of medicines. The ready access to smart phones and the internet should also be applied to the collation and dissemination of medicine prices, thus improving access to information and widening consumer choices.

## Figures and Tables

**Table 1:** Comparison of innovator, lowest priced generic, and international reference prices.

	Product		Unit Price (KShs) *			Price Ratios		
		n	IB	LPG	IRP	IB/LPG	LPG MPR	IB MPR
1	**Omeprazole 20 mg**	16	200	10	1.41	20.0	7.1	141.8
2	**Ranitidine 300 mg**	12	58	11.5	3.75	5.0	3.1	15.5
3	**Furosemide 40 mg**	7	40	3	0.62	13.3	4.8	64.5
4	**Enalapril 10 mg**	8	-	10	4.42	-	2.3	-
5	**Nifedipine 20 mg**	8	82	5	2.33	16.4	2.1	35.2
6	**Hydrochlorothiazide 25 mg**	2	-	5	0.43	-	11.6	-
7	**Atenolol 50 mg**	8	-	10	1.07	-	9.3	-
8	**Paracetamol 500 mg**	16	3.2	1	0.44	3.2	2.3	7.3
9	**Paracetamol 125 mg/5mL syrup (100 mL)**	24	320	50	52.0	6.4	1.0	6.2
10	**Ibuprofen 400 mg**	13	-	1	1.18	-	0.8	-
11	**Diclofenac 50 mg**	13	53	2.8	0.45	18.9	6.2	117.8
12	**Amoxicillin 500 mg**	13	25	1	3.00	25.0	0.3	8.3
13	**Amoxicillin 125 mg/5 mL (100 mL)**	13	320	50	46.0	6.4	1.1	7.0
14	**AmoxiClav 500 mg/125 mg**	17	125	50	16.4	2.5	3.0	7.6
15	**AmoxiClav 250/62.5 mg/5 mL (100 mL)**	18	800	150	521	5.3	0.3	1.5
16	**Sulfamethox./Trimethoprim 400 mg/80 mg**	6	-	5	1.20	-	4.2	-
17	**Sulfamethox./Trimethoprim 200/40 mg/5 mL (100 mL)**	11	400	50	42.0	8.0	1.2	9.5
18	**Ciprofloxacin 500 mg**	15	350	10	3.73	35.0	2.7	93.8
19	**Ceftriaxone 1 g**	18	2670	35	39.8	76.3	0.9	67.1
20	**Clotrimazole cream 20 g**	12	235	40	27.0	5.9	1.5	8.7
21	**Clotrimazole pessary 100 mg**	11	142	17	7.50	8.4	2.3	18.9
22	**Fluconazole 200 mg**	8	130	10	7.03	13.0	1.4	18.5
23	**Aciclovir 400 mg**	5	-	10	8.35	-	1.2	-
24	**Chlorpheniramine 4 mg**	11	30	1	0.20	30.0	5.0	150.0
25	**Cetirizine 10 mg**	11	50	10	1.20	5.0	8.3	41.7
26	**Salbutamol inhaler (200 dose)**	3	350	400	184	0.9	2.2	1.9
27	**Amitriptyline 25 mg**	1	-	2	0.84	-	2.4	-
28	**Artemether/lumefantrine 20 mg/120 mg**	13	17	3	16.2	5.7	0.2	1.0
29	**Metformin 500 mg**	8	14	1	1.50	14.0	0.7	9.3
30	**Glibenclamide 5 mg**	2	15	3	0.57	5.0	5.3	26.3
31	**Metronidazole 400 mg**	8	9	1	1.18	9.0	0.8	7.6
32	**Metronidazole 200 mg/5 mL (100 mL)**	13	600	20	54	30.0	0.4	11.1
33	**Albendazole 400 mg**	16	200	15	7.88	13.3	1.9	25.4
34	**Albendazole 200 mg/5 mL (10 mL)**	11	200	50	32.0	4.0	1.6	6.3
35	**Levonorgestrel 0.75 mg**	7	75	50	22.5	1.5	2.2	3.3

IB = Innovator brand, LPG = Lowest priced generic, IRP = 2015 MSH international reference median price, MPR = Median price ratio i.e., LPG/IRP; * 100 Kenyan Shillings (KShs) ≈ 1 USD. n = number of different generic brands.

**Table 2:** Price variation in therapeutic classes and affordability of standard treatment regimens.

Medicine	LPG Price Per Course	Days Worked for the LPGW to Buy a Treatment Course Assuming Disposable Income		
		100% *	40%	7%
**ANTI-ULCERS**				
Omeprazole 20 mg (1 × 2 tablets daily, 30 days)	300	0.57	1.42	8.13
Ranitidine 300 mg (1 tablet daily, 30 days)	345	0.65	1.64	9.35
**ANTIPROTOZOALS FOR ACUTE AMEBIASIS**				
Metronidazole 400 mg (1 × 3 tablets daily, 5 days)	15	0.03	0.07	0.43
**ANTIBACTERIALS (FOR UPPER RESPIRATORY TRACT INFECTIONS) AND INHALER**				
Ciprofloxacin 500 mg (1 × 2 tablets daily, 7 days)	140	0.27	0.66	3.79
Amoxicillin 500 mg (1 × 3 capsules daily, 7 days)	21	0.04	0.10	0. 57
Amoxicillin/Clavulanic acid 625 mg (1 × 2 tablets daily, 7 days)	700	1.33	3..32	18.97
Ceftriaxone 1000 mg (1g daily, 7 days)	245	0.46	1.16	6.64
Sulfamethox./Trimethoprim 240 mg/5 mL (1 bottle)	400	0.76	1.90	10.84
Salbutamol inhaler 1 mcg/dose (1 inhaler)	400	0.76	1.90	10.84
**ANTIHYPERTENSIVES**				
Hydrochlorthiazide 25 mg (1 × 1 tablet daily, 30 days)	150	0.28	0.71	4.07
Furosemide 40 mg (1 × 1 tablet daily, 30 days)	90	0.17	0.43	2.44
Atenolol 50 mg (1 × 1 tablet daily, 30 days)	300	0.57	1.42	8.13
Enalapril 20 mg (1 × 1 tablet daily, 30 days)	300	0.57	1.42	8.13
Nifedipine 20 mg (1 × 3 tablet daily, 30 days)	150	0.84	2.13	12.21
**HYPOGLYCEMICS**				
Metformin 500 mg (1 × 3 tablets daily, 30 days)	90	0.18	0.42	2.43
Glibenclamide 5 mg (1 × 1 tablet daily, 30 days)	90	0.17	0.43	2.44

* LPGW method. 100 KShs ≈ 1 USD.

**Table 3:** Availability of a basket of 15 medicines in facilities.

Product	Number of Facilities Stocking the Medicine (% of 45 Facilities)
Aciclovir tablets 400 mg	13 (29)
Amitriptyline tablets	8 (18)
Amoxicillin capsules	**30 (67)**
Atenolol tablets	17 (38)
Beclomethasone inhaler	1 (2)
Ceftriaxone injection	**27 (60)**
Ciprofloxacin tablets	**34 (76)**
Enalapril tablets *	20 (44)
Fluoxetine tablets	2 (4)
Glibenclamide tablets	18 (40)
Hydrochlorthiazide tablets	**27 (60)**
Ranitidine tablets	12 (27)
Omeprazole capsules	**30 (67)**
Salbutamol inhaler	19 (42)
Sulfamethoxazole/Trimethoprim suspension	**30 (67)**

* Replaced captopril. Bold figures highlight: >50% availability.
